# The Analysis of Factors Associated with Progression of Isolated Terminal Ileal Lesions

**DOI:** 10.1371/journal.pone.0090797

**Published:** 2014-03-13

**Authors:** Zhang Fangbin, Hao Weiwei, Zhao Wugan, Zheng Cong, Chu Yanjun, Xu Feng

**Affiliations:** 1 Department of Gastroenterology, The First Affiliated Hospital of Zhengzhou University, Zhengzhou, People's Republic of China; 2 Department of Pathology, The First Affiliated Hospital of Zhengzhou University, Zhengzhou, People's Republic of China; University Hospital Llandough, United Kingdom

## Abstract

**Objective:**

To assess the factors associated with the progression of isolated terminal ileal lesions (ITILs) at colonoscopy in Chinese patients.

**Methods:**

Patients diagnosed with ITILs were enrolled. The ileoscopy was performed by two experienced gastroenterologists every 52 weeks. A logistic regression analysis was used to elucidate the factors associated with Crohn's disease (CD) and mucosal healing. A log rank test was used to assess the differences of the cumulative proportion of CD and mucosal healing in different groups at different times.

**Results:**

(1) A total of 34 patients were included and no patient had taken nonsteroidal anti-inflammatory drug in the last 6 months; eight (23.5%) patients had a clinical diagnosis of CD, 14 (41.2%) patients achieved mucosal healing, and 12 (35.3%) patients showed no significant changes in the lesions at last follow-up. (2) The logistic regression analysis showed that only abdominal pain was a factor in the ITIL disease outcomes. (3) The cumulative proportion of CD in the abdominal pain group after 3 years was statistically higher than that in the non-abdominal pain group (42.7% *vs.* 6.2%, *χ^2^* = 10.129, *P* = 0.001). However, the cumulative proportion of mucosal healing in the non-abdominal pain group was statistically higher than that in the abdominal pain group (73.3% *vs.* 5.6%, *χ^2^* = 5.225, *P* = 0.022). (4) The numbers of lesions observed on the initial colonoscopy exams and the initial histologic findings were not related to the ITIL disease outcomes.

**Conclusions:**

Clinical symptoms may be related to ITIL disease outcomes. Patients with abdominal pain had a high likelihood of CD, whereas those without abdominal pain had a high likelihood of mucosal healing.

## Introduction

Ileal intubation is increasingly recognized as being valuable in patients with abdominal pain, diarrhea or bloody stools—particularly where inflammatory bowel disease is a consideration. [1]–[3] This has led to an increased identification of patients (about 0.1–0.3%) who are not easily classified—those with Isolated ileal abnormalities, which we have coined isolated terminal ileal lesions (ITILs). [4], [5] It is likely that a proportion of these patients are suffering from a restricted form or so-called forme fruste of Crohn's disease (CD). Currently, the clinical significance of this entity is unclear. [2], [6], [7].

The present study evaluated the clinical course of patients with ITILs to determine the clinical significance of these lesions.

## Materials and Methods

### Subjects

Patients diagnosed with ITILs at the Gastroenterology clinic of the First Affiliated Hospital of Zhengzhou University Hospital from 2005 to 2010 were actively recruited to this study with the goal of evaluating the natural history of terminal ileal lesions. All patients underwent gastroscopy and double contrast small bowel radiography to evaluate their upper gastrointestinal and small intestinal mucosa.

Subjects were excluded if they fulfilled any of the following criteria: (1) a follow-up of less than 2 years; (2) a history of nonsteroidal anti-inflammatory drug or glucocorticoid use within the 6 months before the study; (3) erosions in other parts of the colon in addition to the terminal ileum; (4) oral or genital ulcerations suggestive of Behçet disease; (5) infectious colitis (except tuberculosis); (6) a prior history of CD or ulcerative colitis; (7) a history of colorectal cancer or surgery; (8) a history of systemic lupus erythematosus or vasculitis; or (9) poor adherence to the study: rejection of colonoscopies during the follow-up.

### Study Design

Clinical data, including sex, age, clinical symptoms and medical records were recorded. The patient information was collected by a clinician. All patients were asked to ingest 2 L of polyethylene glycol solution in the evening before the ileoscopy. The ileoscopy was performed by two experienced gastroenterologists at weeks 0, 52, 104, 156, 208, 260, 312, 364, 416, 468, and 520. Two biopsy specimens were obtained from the most severe area of the lesion in each subject. All of the biopsy specimens were evaluated separately by two experienced pathologists. In cases in which they did not agree on an evaluation, a discussion with a senior gastrointestinal pathologist was employed.

Drug therapy was directed toward the dominant symptoms: a selective smooth-muscle relaxant for abdominal pain, loperamide for diarrhea, and lactulose syrup or polyethylene glycol for constipation. (Table 1) The treatments were withdrawn when the clinical symptoms resolved and reintroduced when the clinical symptoms recurred. Patients with positive occult blood test, mild abdominal discomfort, or surveillance after colorectal polypectomy underwent no any treatment during the total duration of follow-up.

**Table 1:** Possible Drugs for a Dominant Symptom in Patients.

Symptom	Drug	Dose
Abdominal Pain	Pinaverium bromide	50 mg qd to qid ac
	Amitriptyline	Start 25–50 mg hs, then adjust
	Paroxetine hydrochloride	Start 20 mg hs, then adjust
Diarrhea	Loperamide	2–4 mg when necessary/maximum 12 mg/d
	Pinaverium bromide	50 mg qd to qid ac
Constipation	Lactulose syrup	10–20 g bid
	Polyethylene glycol	17 g in 8 oz water qd
	Mosapride	5 mg tid ac

qd, quaque die; bid, bis in die; qid, quater in die; hs, hora somni; ac, ante cibum; oz, ounce.

The symptoms were assessed daily by asking the patient to complete a diary regarding the following: abdominal pain, discomfort, stool frequency, and stool consistency.

Assessment of overall symptom relief was measured weekly. (Table 2) Patients were assessed for symptom recurrence weekly by telephone interview and monthly at a clinic visit.

**Table 2:** Overall Relief Assessment.

Abdominal pain
0 = none (no symptoms)
1 = mild (presence of symptoms but well-tolerated)
2 = moderate (symptoms interfere with but do not prevent normal daily activities such as work and/or sleep)
3 = severe (symptoms prevent normal daily activities such as work and/or sleep)
Stool frequency
the daily number of bowel movements was recorded
Stool consistency [8]
1 = separate hard lumps, like nuts
2 = sausage-like, but lumpy
3 = like a sausage but with cracks in the surface
4 = like a sausage, smooth and soft
5 = soft blobs with clear-cut edges
6 = fluffy pieces with ragged edges, a mushy stool
7 = watery, no solid pieces

The patients recorded the overall clinical symptom relief in a diary by answering ‘yes’ or ‘no’ to the following question: ‘Do you consider that over the past week you have had satisfactory relief from your symptoms?’ ‘Satisfactory’ meant that compared with their typical experience of the disorder in the past, the patient felt that during the past week their clinical symptoms had been alleviated to the extent that they would take medication to maintain that state.

At scheduled doctor visits, response to initial treatment was designated when patients' overall satisfactory relief from symptoms for at least 2 of the 4 previous weeks; those whose overall satisfactory relief from symptoms for less than 2 weeks in the 4 previous weeks were considered nonresponders.

Patients requesting urgent restoration of medication treatment because of recurring/worsening symptoms before or at a scheduled telephone contact during the withdrawal period were considered to have relapsed.

### Statistical Analyses

Statistical analysis was performed using SPSS software, version 16.0 (SPSS Inc., Chicago, Ill, USA). To elucidate the factors associated with developing CD and mucosal healing, a logistic regression analysis was used and results were expressed as odds ratios (OR) with 95% confidence intervals (CI). A Kaplan–Meier plot and a log-rank test were used to visualize and to test differences between CD and mucosal healing in probability of remaining in the study. A *χ^2^* test was used to compare the endoscopic findings, pathologic findings on the initial colonoscopy exams and disease outcomes of ITILs. A *P*-value less than 0.05 was considered to be significant.

### Ethical Considerations

The ethics committee of the participating center approved the study (reference numbers: 20050401GS-7 (University of Zhengzhou)). The study was conducted according to the principles expressed in the Declaration of Helsinki. Written informed consent was obtained from all participants at recruitment.

## Results

### Subject Characteristics

During the study period, 32,197 patients underwent colonoscopic examinations at the Gastrointestinal Endoscopy Center. Of these, 43 were assigned an endoscopic diagnostic code for ITILs. Of these 43 patients, seven patients (16.3%) with a follow-up of less than 2 years and two patients (4.7%) with poor compliance were excluded, the data of these 9 patients wasn't analyzed in this study; the remaining 34 patients (79.1%) met the study criteria. These 34 patients including in this study have no a history of nonsteroidal anti-inflammatory drug (such as aspirin) or glucocorticoid use within the 6 months before the study. These 34 patients including in this study have no family history of IBD.

The patient sample consisted of 19 men and 15 women with a mean age of 33.3±11.1 years (range 22–62 years), and the mean follow-up was 3 years (range 2–5 years). The indications for colonoscopy included abdominal pain in 10 (29.4%) patients, stools >3 times daily in nine patients (26.5%), abdominal pain and diarrhea in four patients (11.8%), abdominal pain and constipation in two patients (5.9%), positive occult blood test in two patients (5.9%), mild abdominal discomfort in two patients (5.9%), constipation in one patient (2.9%), diarrhea in one patient (2.9%), and surveillance after colorectal polypectomy in three patients (8.8%). Table 3 shows the demographic characteristics of the 34 patients.

**Table 3:** The Demographic Characteristics of the 34 Patients.

Sex (male∶female)	19∶15
Mean age at diagnosis (y)	33±11 (22–62)
Duration of follow-up (y)	3 (2–5)
Symptoms	
Abdominal pain	10 (29%)
Stools >3 times daily	9 (26%)
Abdominal pain and diarrhea	4 (12%)
Abdominal pain and constipation	2 (6%)
Positive occult blood test	2 (6%)
Mild abdominal discomfort	2 (6%)
Constipation	1 (3%)
Diarrhea	1 (3%)
Surveillance after colorectal polypectomy	3 (9%)
Colonoscopy	3±1 (2–5)

According to the Rome III criteria, [9] two of the 34 patients (5.9%) with abdominal pain and diarrhea and two of the 34 patients (5.9%) with abdominal pain and constipation met criteria for irritable bowel syndrome (IBS), two of the 34 patients (5.9%) with abdominal discomfort were considered functional bloating, one of the 34 patients (2.9%) with constipation was considered functional constipation, one of the 34 patients (2.9%) with diarrhea was considered functional diarrhea, two of the 34 patients (5.9%) with abdominal pain and diarrhea were considered unspecified functional bowel disorder.

### Clinical Findings

Of the 34 patients, eight (23.5%) were eventually diagnosed with CD on follow-up, 14 (41.2%) achieved mucosal healing, and 12 (35.3%) showed no significant changes in the lesions. Of the 31 patients categorized as symptomatic (symptom onset at least 3 days per month in the last 3 months), eight (26%) had a clinical diagnosis of CD during the follow-up; the mean interval between the first visit and the appearance of typical CD symptoms was 2.0 years (range 1.0–4.0 years). Of the three patients categorized as asymptomatic (without symptom or symptom onset less than 3 days per month in the last 3 months), 2 (66.7%) achieved mucosal healing, and one (33.3%) showed no significant changes in the lesions.

Of the 31 symptomatic patients, 29 (93.5%, 29/31) underwent drug therapy based on their dominant symptoms, 26 (89.7%, 26/29) attained recovery during the initial treatment period, 23 (88.5%, 23/26) experienced symptoms relapse during the withdrawal period, and 20 (87.0%, 20/23) attained recovery during the retreatment period.

Of the 12 patients who showed no significant changes in the lesions, all attained recovery during the initial treatment period, 10 (83.3%, 10/12) experienced symptom relapse during the withdrawal period, and nine (90.0%, 9/10) attained recovery during the retreatment period.

### Endoscopic and Pathologic Findings

Normal mucosa was observed on the gastroscopies and radiological exams in the 34 patients.

**Figure 1:**
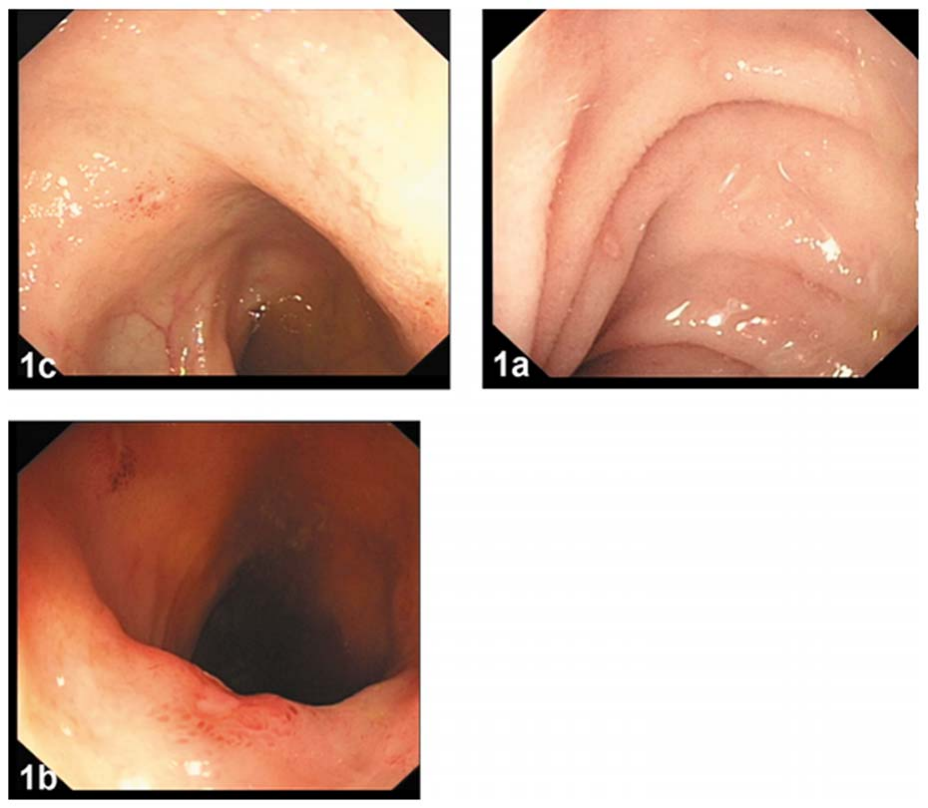
Initial colonoscopic images in the 34 patients. Erosions surrounded by normal mucosa (1a), edematous mucosa (1b), and covered by thin or faint exudates (1a, 1b, and 1c).

All of the 34 patients underwent ileoscopy. On the initial colonoscopy exams, each case demonstrated one to several discrete, focal erosions within an otherwise normal small bowel mucosa. The erosions were located less than 5 cm proximal to the ileocecal valve. Most of the erosions were surrounded by normal or edematous mucosa and were covered by thin or faint exudates. (Fig. 1a, 1b, and 1c) The size of the erosions was estimated to be 2 to 3 mm. Of the 34 patients, 10 patients (29.4%) had a single erosion, and 24 patients (70.6%) had several erosions.

**Figure 2:**
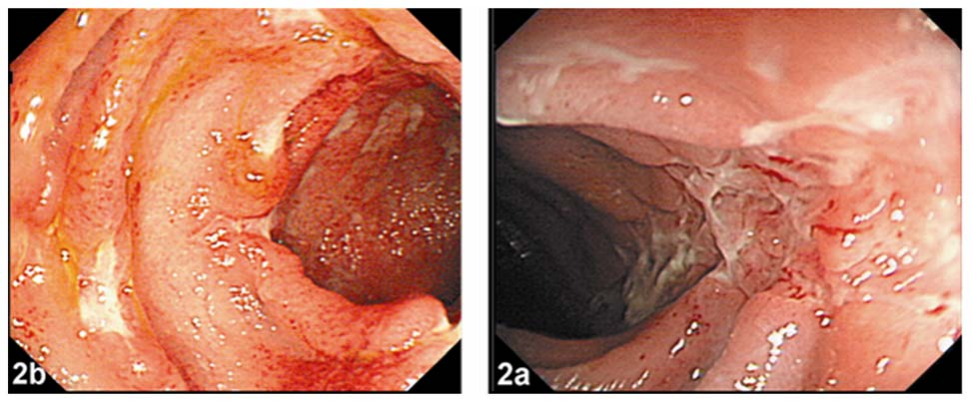
Colonoscopic images in the patients who developed CD. Typical longitudinal ulcerations of CD covered with mucous exudates (2a), and irregular ulcerations existed of CD covered with mucous exudates (2b). CD, Crohn's disease.

Of the eight patients with a clinical diagnosis of CD, typical longitudinal ulcerations existed in seven patients, irregular ulcerations existed in one patient (Fig. 2a and 2b), narrowing or stricture on imaging in four patients, perianal disease in one patient, and multiple episodes of small bowel obstruction in one patient.

The initial histologic findings in the patients included mucosal edema (7), erosions (8), acute or chronic inflammatory cells (11) and architectural alterations (3). The 8 patients with a final diagnosis of CD had non-caseating granulomas (3), architectural alterations (9) cryptitis (3), or focal chronic (lymphocytes and plasma cells) inflammation (7).

All of the patients responded to inflammatory bowel disease (IBD) treatments (1 patient was treated with infliximab and azathioprine, two patients were treated with azathioprine, and five patients were treated with 5-amino salicylic acid) and were still on maintenance therapy for CD at the most recent follow-up.

### Clinical Symptoms and Outcomes

We conducted a logistic regression analysis to assess the factors associated with progression of isolated terminal ileal lesions in the 34 patients. The independent variables were age, sex, clinical symptoms, and duration of follow-up; the dependent variables were developing CD and mucosal healing. In this regression model, only abdominal pain was significantly associated with developing CD and mucosal healing (developing CD: OR 13.1 95% CI, 1.4–124.5, *P* = 0.025; mucosal healing OR = 38.1 95% CI, 3.5–414.0, *P* = 0.003).

The 34 patients were divided into an abdominal pain group and an non-abdominal pain group based on their clinical symptoms; the abdominal pain group contained 16 patients (10 patients with abdominal pain, four patients with abdominal pain and diarrhea, and two patients with abdominal pain and constipation), and the non-abdominal pain group contained 18 patients (nine patients with stools >3 times daily, two patients with positive occult blood tests, two patients with mild abdominal discomfort, three patients with surveillance after colorectal polypectomy, one patient with constipation, and one patient with diarrhea). In the abdominal pain group, seven patients were eventually diagnosed with CD on follow-up, Including two patients with IBS. The median interval between the first visit and the presentation of typical CD symptoms was 2 years (range 1–4 years). In the remaining nine patients with abdominal pain, eight showed no significant changes compared with their initial colonoscopic findings, and one patient achieved mucosal healing after anti-tuberculosis therapy for 9 months. In the non-abdominal pain group, 13 patients achieved mucosal healing. The median interval between the first visit and mucosal healing was 3 years (range 1–5 years). In the remaining five patients without abdominal pain, four showed no significant changes, and one patient with a positive occult blood test developed CD in the 3^rd^ year of follow-up.

**Figure 3:**
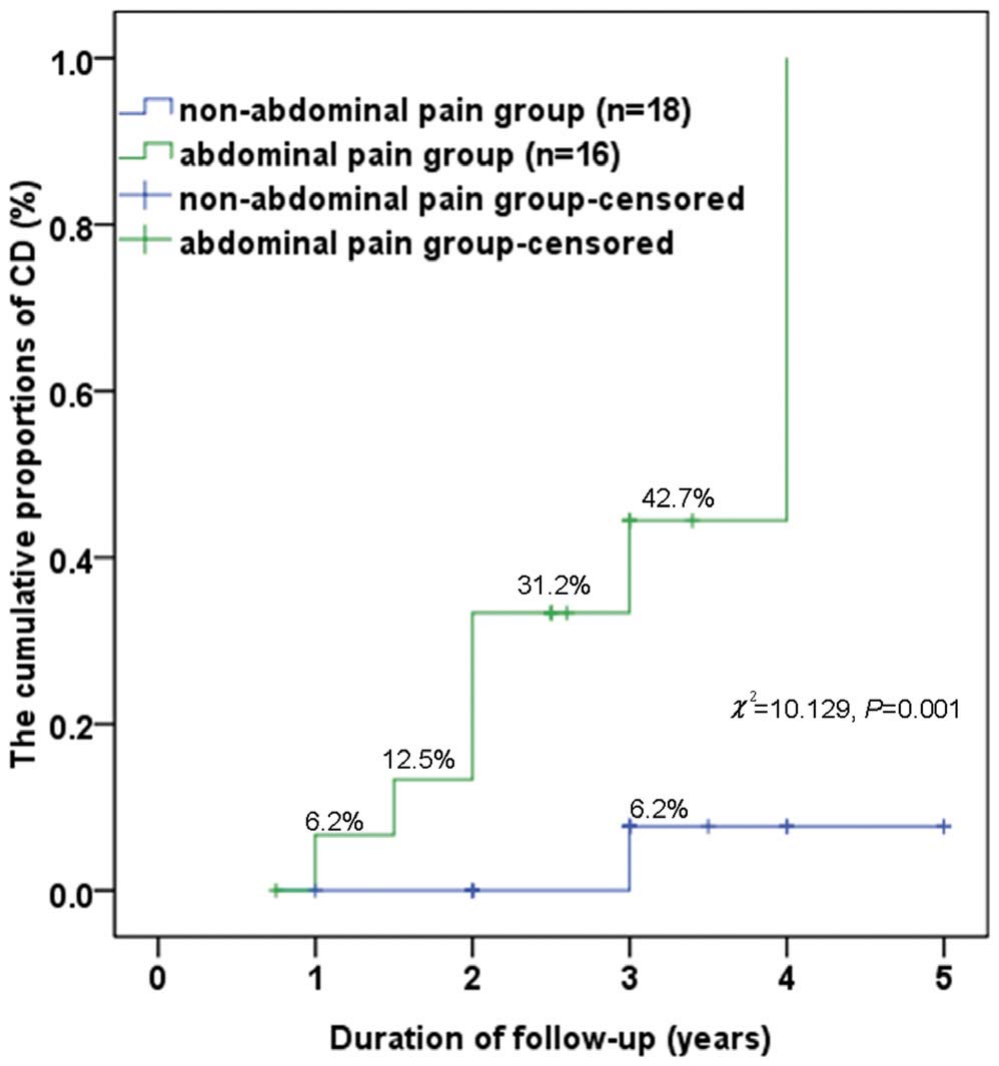
The cumulative proportions of CD in the 34 patients. The cumulative proportions of CD in the abdominal pain group in the 1^st^, 2^nd^, and 3^rd^ years of follow-up were 6%, 31% and 43%, respectively, and the proportions in the non-abdominal pain group were 0, 0, and 6%, respectively. Moreover, the cumulative proportion of CD in the abdominal pain group after 3 years was statistically higher than that in the non-abdominal pain group (43% *vs.* 6%, *χ^2^* = 10.129, *P* = 0.001). CD, Crohn's disease.

**Figure 4:**
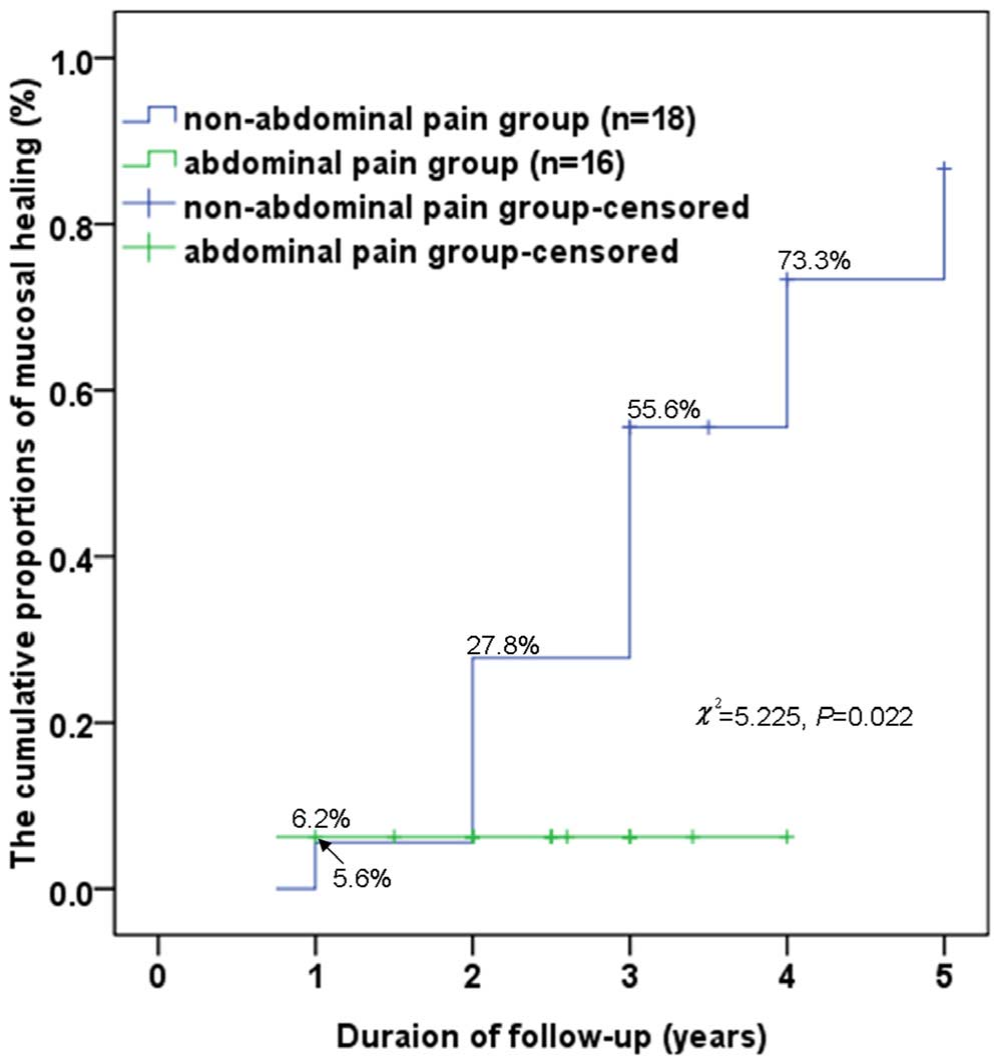
The cumulative proportions of mucosal healing in the 34 patients. The cumulative proportions of mucosal healing in the non-abdominal pain group in the 1^st^, 2^nd^, 3^rd^, and 4^th^ years of follow-up were 6%, 28%, 56% and 73%, respectively, and the proportions in the abdominal pain group were 6%, 6%, 6% and 6%, respectively. Moreover, the cumulative proportion of mucosal healing in the non-abdominal pain group after 4 years was statistically higher than that in the abdominal pain group (73% *vs.* 6%, *χ^2^* = 5.225, *P* = 0.022).

Of the 34 patients, sex and age were not significantly different between the abdominal pain and non-abdominal pain groups. The Kaplan–Meier method showed that the cumulative proportions of CD in the abdominal pain group in the 1^st^, 2^nd^, and 3^rd^ years of follow-up were 6.2%, 31.2% and 42.7%, respectively, whereas in the non-abdominal pain group, the proportions were 0, 0, and 6.2%, respectively. The log-rank test showed that the cumulative proportion of CD in the abdominal pain group after 3 years was statistically higher than that in the non-abdominal pain group (42.7% *vs.* 6.2%, *χ^2^* = 10.129, *P* = 0.001). (Fig. 3) The cumulative proportions of mucosal healing in the non-abdominal pain group in the 1^st^, 2^nd^, 3^rd^, and 4^th^ years of follow-up were 6.2%, 27.8%, 55.6% and 73.3%, respectively, whereas the proportions in the abdominal pain group were 5.6%, 5.6%, 5.6% and 5.6%, respectively. The cumulative proportion of mucosal healing in the non-abdominal pain group after 4 years was statistically higher than that in the abdominal pain group (73% *vs.* 6%, *χ^2^* = 5.225, *P* = 0.022). (Fig. 4)

### Endoscopic Findings, Pathologic Findings and Outcomes

Next, we analyzed the association between the initial endoscopic findings and ITIL disease outcomes. We found that the numbers of lesions on the initial colonoscopy exams did not differ in the patients who were eventually diagnosed with CD on follow-up, achieved mucosal healing, and showed no significant changes in the lesions (2 (1–3) *vs.* 2.5 (1–3) *vs.* 2.5 (1–3), *χ^2^* = 1.252, *P* = 0.535). Similarly, the initial histologic findings did not differ in these three groups (the proportion of patients with mucosal edema 25.0% (2/8) *vs.* 21.4% (3/14) *vs.*16.7% (2/12), *χ^2^* = 0.427, *P* = 1.000; the proportion of patients with erosions 12.5% (1/8) *vs.* 21.4% (3/14) *vs.* 33.3% (4/12), *χ^2^* = 1.144, *P* = 0.678; the proportion of patients with acute or chronic inflammatory cells 25.0% (2/8) *vs.* 50.0% (7/14) *vs.*16.7% (2/12), *χ^2^* = 3.304, *P* = 0.207; and the proportion of patients with architectural alterations 12.5% (1/8) *vs.* 0 (0/14) *vs.* 16.7% (2/12), *χ^2^* = 2.543, *P* = 0.317).

## Discussion

Through a prospective study, we aimed to assess the factors associated with progression of ITILs in 34 Chinese patients.

ITILs are not uncommonly seen during routine screening colonoscopy, the frequency was about 0.1–0.3% and 0.1% (34/32,197) in this study. [4], [5] The clinical significance of the ITILs is unclear. Goldstein et al. reported that eight of 28 patients (28.6%) developed typical CD at an average interval of 3.6 years. [7] Then, Courville et al. reported that 10 of 29 patients (34.5%) developed typical CD at an average interval of 2.2 to 12.6 years. [10] A recent study by Chang et al. reported that 1 of 93 patients (1.1%) developed typical CD at an average interval of 29.9 months. [4] Conversely, Lengeling et al. reported that 40 patients identified with “ulcerative ileitis” at ileocolonoscopy had no specific disease process development in a median follow-up of 3.2 years. [5] In this study, 23.5% of the patients were eventually diagnosed with CD on follow-up, and 41.2% of the patients achieved mucosal healing. The lower probability of achieving mucosal healing in this study could have been the result of the patients with different clinical symptoms, different follow-up lengths, and racial differences.

Aphthoid or small erosions have been considered one of the earliest manifestations of CD. Two previous studies showed that 44% (4/9) and 50% (5/10) of patients with aphthous-type CD later developed typical CD. [11], [12] More recent studies have shown that the disease outcomes of ITILs are related to the clinical symptoms of patients. A study by Goldstein et al. reported that all 8 patients (29%) with ITILs who had developed CD on follow-up presented with abdominal pain, mucus-rich, blood-tinged stools; irregular bowel function with intermittent constipation and diarrhea; and low-level systemic malaise. [7] Recently, a study by Courville et al. reported that 10 of 15 (66.7%) symptomatic patients, and 0 of 14 asymptomatic patients had developed CD at the most recent follow-up. [10] Our findings are similar in those patients undergoing colonoscopy for symptoms; eight of 31 (26%) symptomatic patients and zero of three asymptomatic patients had developed CD during the follow-up. We conducted a logistic regression analysis and found that only abdominal pain was significantly associated with developing CD. Further analysis showed that the cumulative proportion of CD in the abdominal pain group after 3 years was statistically higher than that in the non-abdominal pain group.

Should patients with ITILs be treated? Two studies reported that isolated terminal ileal ulcerations completely resolved without any treatment on follow-up colonoscopy in 66.7% of asymptomatic patients (four of six patients and 62 of 93 patients, respectively). [4], [10] In this present study, two of the three (66.7%) asymptomatic patients completely resolved without inflammatory bowel disease-related treatment. We conducted a logistic regression analysis and found that only abdominal pain was significantly associated with mucosal healing. Further analysis showed that the cumulative proportion of mucosal healing in the non-abdominal pain group was statistically higher than that in the abdominal pain group. Our findings suggest that these patients in the non-abdominal pain group do not warrant any inflammatory bowel disease-related treatment, and a wait and watch approach seems to be the most prudent at the present time.

A study by Courville et al. reported that the endoscopic and histopathological findings in patients with asymptomatic ileitis closely mimicked those observed in CD, but these patients did not progress to overt CD on long-term follow-up. [10] A recent study by Chang et al. reported that the proportion of patients with ulcerations <5 mm in diameter was not significantly different between the 60 patients showing isolated terminal ileal ulcerations resolution without treatment and the 31 patients with continued isolated terminal ileal ulcerations on follow-up. [4] Similar to these reports, we found that the numbers of lesions on the initial colonoscopy exams were not related to the ITIL disease outcomes. Similarly, the initial histologic findings were not related to the ITIL disease outcomes. Thus, the endoscopic or histopathological findings may not be useful in predicting the disease outcomes in patients with ITILs.

Irregular bowel movements, pain or bloating may coexist in a considerable number of IBD patients, which were present in 35.4–46% of CD patients and 37.5% (3/8) in this study. [13], [14] There is uncertainty as to the aetiology of these apparent functional symptoms in IBD patients. Genetic factors, disordered gut permeability, psychosocial stress, or a persistent increase in TNF-α or mucosal pain receptor in colonic mucosa may contribute to these symptoms. [14]–[21] A previous study by Keohane et al. found that faecal calprotectin levels were helpful to identify CD patients with IBS-type symptoms. [22] Then, a study by Berrill et al. found a conflicting result. [23] However, a systematic diagnostic approach is required to assess IBD patients with IBS-type symptoms as sub-clinical inflammation may play a role in a proportion of cases. [23]

Calprotectin is an abundant calcium-binding protein that is derived predominantly from neutrophils and, to a lesser extent, from monocytes and reactive macrophages. [24] The presence of calprotectin in faeces is directly proportional to neutrophil migration towards the intestinal tract. [25]–[27] Two studies by Tibble and Carroccio et al. found that approximately half of patients with active celiac disease had elevated fecal calprotectin concentrations. [28], [29] Another study by Berni et al examined a total of 38 children with celiac disease. They found that those patients with active celiac disease had elevated fecal calprotectin concentrations compared with healthy control subjects and that there was a trend toward normal values after 4 weeks of an exclusion diet. [30] However, the specificity for the diagnosis of ITILs should be low, as several diseases such IBD, microscopic colitis, allergic colitis, colorectal neoplasia, gastrointestinal infection and chronic diarrhea can also increase faecal calprotectin. [29], [31]–[34] High concentration of calprotectin in faeces is a strong argument to carry out a colonoscopy in order to rule out the presence of IBD or other organic pathologies. Maybe faecal calprotectin is useful during the follow-up on ITILs.

In conclusion, the results of this study suggest that the ITIL disease outcomes are related to the clinical symptoms. ITILs are likely to develop into CD in patients with abdominal pain but are likely to resolve in patients without abdominal pain. It should be noted that all patients underwent gastroscopy and double contrast small bowel radiography instead of MRI, or videocapsule to evaluate their upper gastrointestinal and small intestinal mucosa, so subtle small bowel lesions may be missed diagnosis in these patients. Our study is limited by the small number of patients. Larger prospective studies are needed to confirm these initial findings. It remains unclear whether ITILs should be treated with 5-aminosalicylates or corticosteroids in patients with abdominal pain. Thus, further randomized, double-blind, placebo-controlled studies in large cohorts of subjects are needed to verify this question.
